# Autoradiographic analysis of internal plutonium radiation exposure in Nagasaki atomic bomb victims

**DOI:** 10.1016/j.heliyon.2018.e00666

**Published:** 2018-06-29

**Authors:** Kazuko Shichijo, Toshihiro Takatsuji, Manabu Fukumoto, Masahiro Nakashima, Mutsumi M. Matsuyama, Ichiro Sekine

**Affiliations:** a Division of Tumor and Diagnostic Pathology, Atomic Bomb Disease Institute, Nagasaki University, Nagasaki, Japan; b Faculty of Environmental Science, Nagasaki University, Nagasaki, Japan; c Department of Molecular Pathology, Tokyo Medical University, Tokyo, Japan; d Tissue and Histopathology Section, Atomic Bomb Disease Institute, Nagasaki University, Nagasaki, Japan

**Keywords:** Biophysics, Pathology, Nuclear medicine

## Abstract

**Background:**

Radiation doses received by Hiroshima and Nagasaki atomic bomb survivors has been evaluated from data related only to external exposure because there was no reliable evidence for internal exposure in atomic bomb victims. However, we assumed that the contribution of internal exposure cannot be ruled out.

**Methods:**

Autoradiography was carried out with the 70-year old paraffin-embedded specimens taken from Nagasaki atomic bomb victims who died within 5 months after the bombing. After exposure to photo emulsion for 6 months alpha-tracks were revealed in the specimens. We confirmed the alpha-tracks were emitted from deposited plutonium (Pu) in reference to the track length of the 8.787 MeV alpha-particle of thorium series from Polonium-212. Radioactivity concentration of Pu was obtained by counting alpha-tracks. The absorbed dose of each cell nucleus penetrated by an alpha-particle was estimated by calculating the absorbed energy from the particle.

**Results:**

Using old paraffin embedded sections processed about 70 years ago, we demonstrated for the first time that conditions in the aftermath of the bombing led to internal exposure to alpha-particles emitted from Pu, the fissile material of the Nagasaki atomic bomb. Dose rate of internal exposure was higher in the victims exposed outdoors than those indoors. Radioactivity concentration was relatively uniform among organs examined in a victim.

**Conclusion:**

Pu was deposited in the bodies of the Nagasaki A-bomb victims presumably via various routes. Organ dose from Pu of the Nagasaki A-bomb victims studied was during their surviving period, which is lower compared with external exposure. However, the impact to the individual cell nucleus by a single alpha-particle might not be negligible, It would be meaningful; to analyze the relationship of the impact of internal exposure at the cellular level and organ dose. The 70-year old pathological specimens utilized in our study are an invaluable source for understanding internal radiation exposure and are crucial in elucidating experimentally unreproducible phenomena.

## 1 Introduction

A uranium type nuclear bomb was used in the attack on Hiroshima city on August 6, 1945. A plutonium (Pu) type nuclear bomb was dropped on Nagasaki city on August 9, 3 days later. Different from naturally existing uranium, almost all plutonium is artificial. Pathological characteristics of atomic-bomb (A-bomb) survivors (hibakusha) deceased within 1 year from the bombing in Hiroshima and Nagasaki have been described (Liebow et al., 1949). However, the dose and the effect of residual radiation, which are controversial, are not addressed (Kerr et al., 2015). Acute radiation syndrome occurred in hibakusha may be augmented by residual radionuclides with short half-life (Sutou, 2017). Nevertheless, both acute radiation syndrome and long-term effects are reportedly not associated with the black rain which fell shortly after the bombing (Sakata et al., 2014; Ozasa et al., 2016). Today about 200,000 A-bomb survivors are alive (Japanese Ministry of Health, Labour, and Welfare) and among them, about 53,000 are living in Nagasaki prefecture, Japan (http://www.mhlw.go.jp/bunya/kenkou/genbaku09/15b.html browsed on Dec. 11, 2017). Radiation risks to each organ have been evaluated using data collected from survivors and dose data of Dosimetry System 2002, which addresses only external dose without internal dose (Cullings et al., 2006). Compared with Hiroshima, a theoretical evaluation revealed a small but significant increase in cancer risk at low doses in Nagasaki hibakusha, suggesting the existence of factors characteristic to Nagasaki such as internal exposure to ^239^Pu (Sasaki et al., 2014). However, it is impossible to ascertain the true condition of the internal exposure 70 years after the event using A-bomb survivors' specimens, because of decrease of radioactivity owing to metabolism and excretion in human tissues (Bair and Thompson, 1974). We have some pathological specimens preserved in paraffin blocks of Nagasaki A-bomb victims who were exposed within 1 km of the hypocenter and died of acute radiation sickness within 5 months (Liebow et al., 1949). Assuming residual radioactive materials attributable to the A-bomb have existed in the autopsied specimens, the radioactive materials would have decreased only by physical disintegration and not by biological excretion. These prompted us to attempt to estimate internal exposure of hibakusha using pathological specimens. The specimens from Nagasaki A-bomb victims are unique in the fact that Pu is clearly an A-bomb material if deposited in their organs. Any internal deposition of alpha-emitters in Hiroshima A-bomb victims cannot be conclusively connected to the bomb material. Because uranium concentration in the soil of Hiroshima is higher than elsewhere in Japan (Takada et al., 1983), naturally occurring uranium cannot be ruled out in the Hiroshima specimens.

Here, we performed autoradiography using the paraffin-embedded specimens from Nagasaki A-bomb victims who died within 5 months after the bombing. We hereafter call them the Nagasaki victims. We identified the alpha emitters as ^239^Pu in the specimens of the Nagasaki victims dissected for pathological investigation 70 years ago.

## 2 Results

### 2.1 Autoradiographs of plutonium alpha in autopsy tissue samples from A-bomb cases

**Fig. 1:**
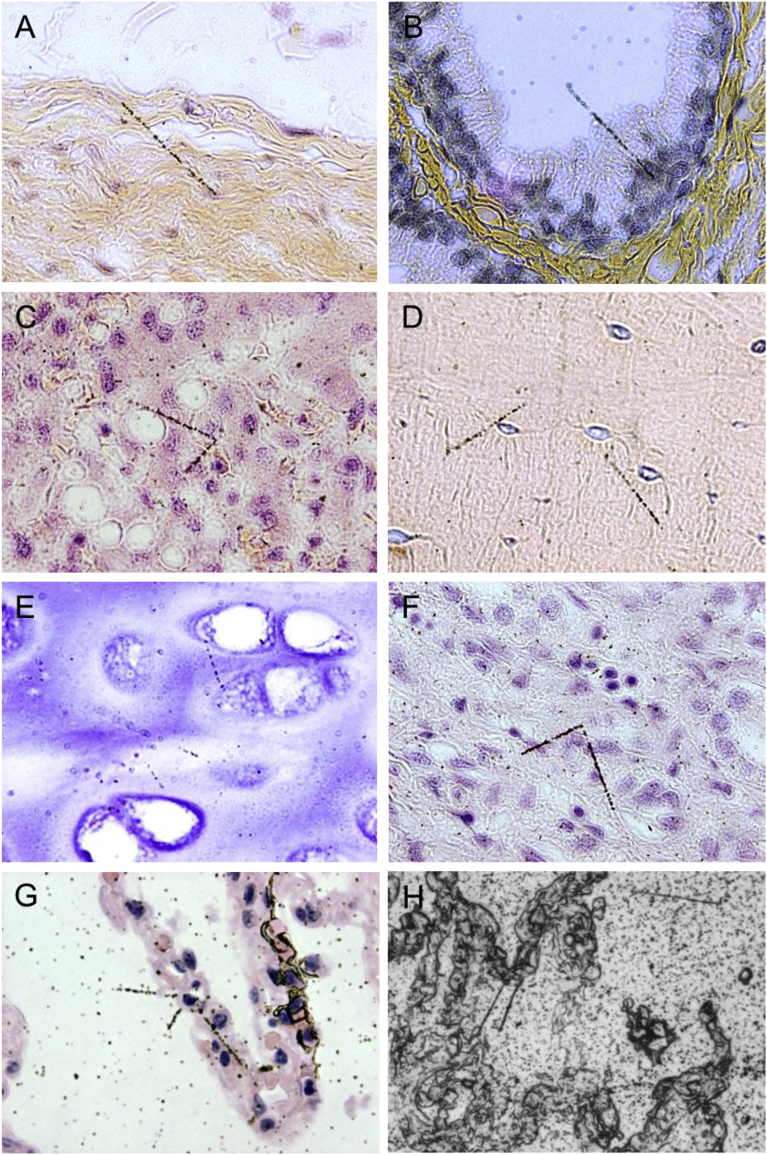
Autoradiographs of plutonium alpha in autopsy tissue samples from the Nagasaki A-bomb cases. Case 1 bladder (A), Case 2 prostate (B), Case 3 liver (C), Case 5 bone (D), Case 5 cartilage of trachea (E), Case 6 kidney (F) and Case 6 lung (G) by photo emulsion dipping method, and Case 6 lung by nuclear emulsion contact method (H).

Alpha-tracks in the paraffin-embedded specimens were observed by autoradiography. One to three alpha-tracks radiating from a point or several points per slide were observed in the organs of the Nagasaki victims, indicating that only a small part of cells within the range of alpha-tracks were exposed. Alpha-tracks were not concentrated in macrophages but were emitted from parenchymal cells in the organs of Nagasaki victims (Fig. 1). In the Thorotrast laden liver, many alpha-tracks were observed in a radial pattern starting from a Thorotrast conglomerate.

### 2.2 Differential and cumulative probability distribution of observed alpha track length

**Fig. 2:**
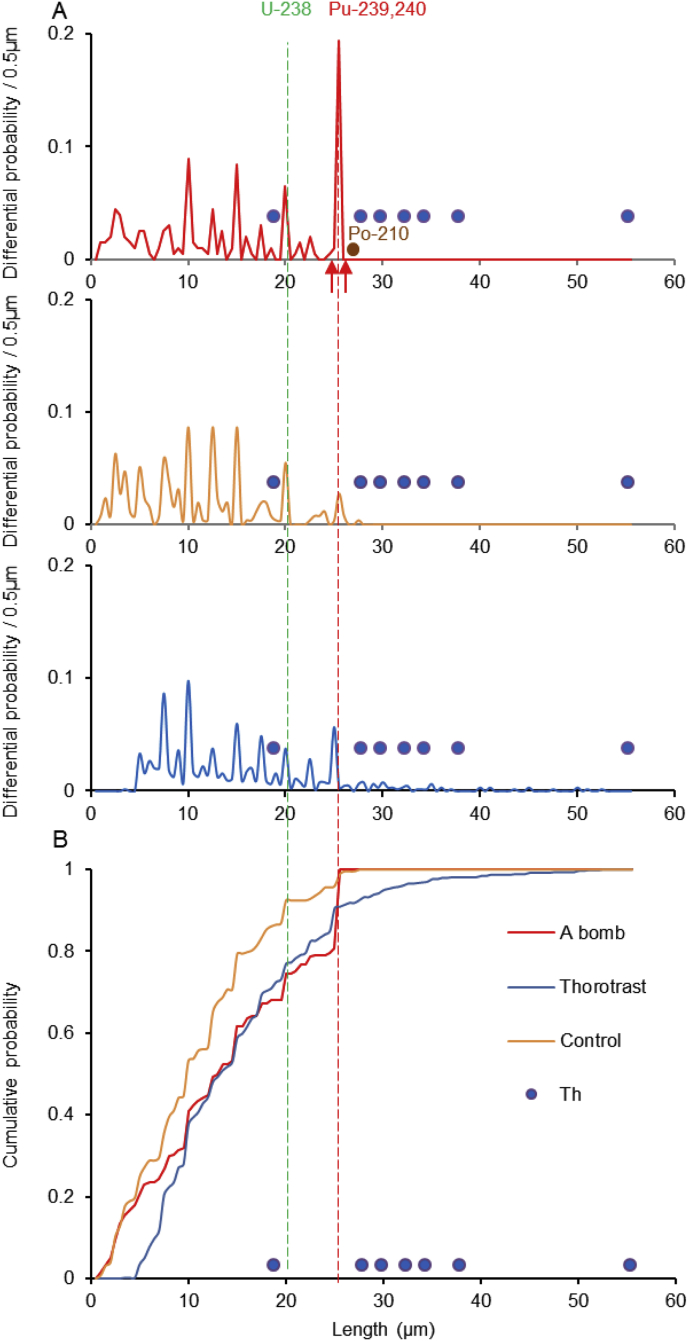
Differential (A) and cumulative (B) probability distribution of observed alpha track length. The numerical data are listed in Table 2, which is the pooled data of all cases. Red arrows indicate the range of the peak area that alpha-tracks are counted as N in Eq. (8).

The probability distribution of the observed track length was calculated from the geometrical conditions of alpha-particles emitted from a tissue specimen between the slide glass and the emulsion layer. From our calculation, the maximum observable track length of the alpha-particle was 25.5 μm, which is close to 5.106–5.157 MeV alpha-particles of ^239^Pu. A sharp peak of the differential probability at 25.5 μm was observed in the Nagasaki victims, but not in the controls or the Thorotrast patient (Fig. 2A). The increase of cumulative probability up to 25.5 μm, was steeper in the Nagasaki victims than the controls or the Thorotrast patient (Fig. 2B). As the origin of alpha-tracks observed in the Nagasaki victims, Thorium-series nuclides could be ruled out by the probability distribution pattern of alpha-tracks. As another possible origin of the alpha-particles in the controls, ^210^Po, a naturally occurring daughter isotope of ^210^Pb, with a 138-day physical half-life emits 5.304 MeV alpha-particles. In order to eliminate ^210^Po as the possible origin we made an autoradiography of the top soil from the common environment containing a large quantity of ^210^Po. The length of alpha-tracks from ^210^Po was clearly distinguished from that of ^239,240^Pu.

### 2.3 Pu activity in the specimens from A-bomb cases

**Fig. 3:**
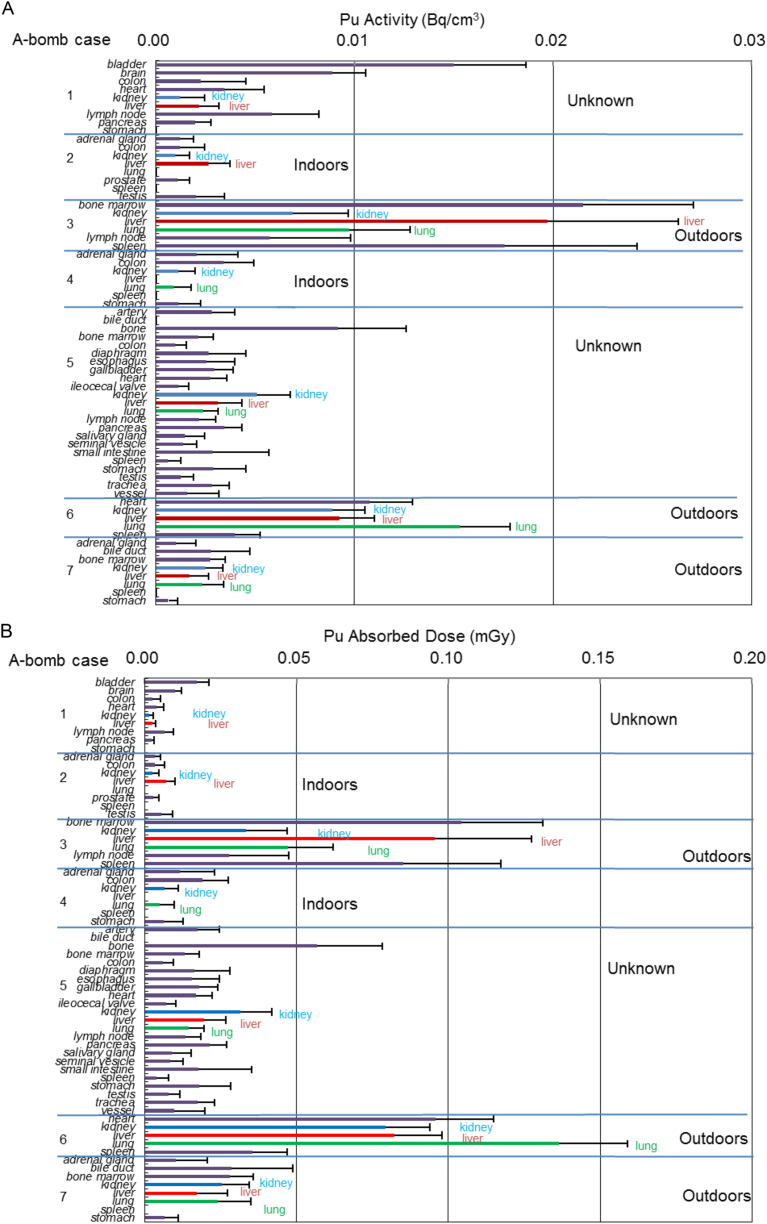
(A) Pu activity in the specimen from A-bomb cases. Activity was calculated according to Eq. (8) using N listed in Table 3. Error bars indicate standard errors of means (SEM) from stochastic variation of the number of alpha particle emissions. (B) Pu absorbed dose during the surviving period of A-bomb cases. Control. 1; liver, lung, spleen, striated muscle, thyroid, 2; lung, 3; kidney, liver, lung, pancreas, spleen, 4; bladder, heart, kidney, liver, lung, spleen, 5; heart, lung, spleen, 6; kidney, lung, spleen, 7; heart, kidney, liver, spleen. No activity (A) or no absorbed dose (B) form Pu was detectable in the above organs examined of controls.

Radioactivity concentration of Pu in various organs was calculated from the density of alpha-tracks and is presented in Fig. 3A. The highest radioactivity concentration in individual organs was observed in Nagasaki case 3 who was exposed within 0.5 km ground distance from the hypocenter, outdoors in the open and died on the 68th day. Among the Nagasaki victims, relatively low radioactivity concentration of individual organs was observed in indoor cases such as Nagasaki case 2 (0.8 km from the hypocenter and died on the 38th day) and case 4 (0.5 km and the 78th day). No radioactivity form Pu was detected in tissues from control subjects. In the Nagasaki victims, radioactivity concentration was not so much different among the organs of the same individual. In Nagasaki case 1, Pu deposit was the highest in the bladder and was relatively high in the lymph node. Interestingly, a significant amount of Pu radioactivity was also observed in the brain of Nagasaki case 1.

The highest absorbed dose rate from Pu for bone marrow (Nagasaki case 3) was 0.560 mGy/y and the accumulated dose during her surviving period was 0.104 mGy. The highest organ absorbed dose rate from Pu for the lung (Nagasaki case 6) was 0.399 mGy/y and the accumulated dose was at most 0.137 mGy during the surviving period (Fig. 3B). Assuming that Pu distribution was unchanged but taking biological excretion of Pu into account (biological half-life 50 years), the accumulated dose for 50 years was estimated at most 20.2 mGy (bone marrow of Nagasaki case 3). Assuming that the cell nucleus is spheroid and a single particle traversed along the long axis, nuclear dose was calculated. The dose could be well approximated by the length of the short axis and the energy of an alpha-particle at the center of the nucleus. We put the alpha-energy at the center of the nucleus at about 1.0 MeV for dose maximum, and the short axis was 3.9 μm for vascular endothelial cells and 6.7 μm for liver parenchymal cells. Absorbed doses of the nucleus of a vascular endothelial cell and a liver parenchymal cell hit by a single alpha-particle were 3.89 Gy and 1.29 Gy respectively.

## 3 Discussion

Almost all fission products and unreacted A-bomb materials were generally assumed to have been swept into the stratosphere. However, we thought that a substantial portion of the unreacted fissile material was likely combined with the debris and dust and fell down. Hibakusha breathing in the dust would have been exposed internally. The nuclear ‘death ash’ would have adhered to the skin. They also would have drunk radioactivity-contaminated water. Nuclides with a short half-life had already disintegrated to a level undetectable by autoradiography. The nuclear material of the Nagasaki A-bomb, ^239^Pu, has a long effective half-life (24,110-year physical half-life and, 50 years biological half-life (Human Health Fact Sheet, 2007)). However, it was reported that radioactivity concentration of Pu even 24 years after the bombing is 0.0175 ± 0.0010 Bq/g in soil of Nishiyama area 2.8 km ground distance from the hypocenter (Sakanoue and Tsuji, 1971). These findings prompted us to investigate the possibility of internal exposure of Nagasaki hibakusha, which is a neglected aspect in the hibakusha study. In this study we performed autoradiography using paraffin-embedded specimens of the Nagasaki victims about 70 years after autopsy. The controls were dissected in hospitals 30 km or 130 km distant from Nagasaki city, not exposed to Nagasaki A-bomb fallout. The source of radioactivity observed in the controls was identified as the Thorium-series and Uranium-series nuclei by the track length distribution. The probability distribution pattern of alpha-track length observed in the Nagasaki victims was apparently consistent with the characteristics of ^239,240^Pu, but not in the controls. Pu radioactivity concentration in the organs of Japanese affected by the global fallout during 1970–1981 is at most 71 × 10^−6^ Bq/g (Taylor, 1995), which is definitely undetectable by the method of this study. We found here, for the first time, ^239^Pu internal deposition in specimens of the Nagasaki victims within 1 km of ground distance from the hypocenter. Since radioactivity of ^239^Pu remaining in the Nagasaki victims was subjected only to physical decay from the day of autopsy to that of autoradiography, that is, 70 years after the bombing. This indicates that Pu radioactivity detected in this study reflects organ exposure at the time of the bombing.

Experimental inhalation of PuO_2_ aerosols into beagle dogs revealed long time retention of Pu in the lung. About 9% of total alveolar deposited Pu is transferred to hilar lymph nodes by 1 year and starts to be detectable in abdominal lymph nodes around 2 years after the inhalation (Bair and Thompson, 1974). After intravascular administration of Pu citrate in the human body, radioactivity concentration is found in the bone marrow, the liver, the bone, the spleen, the kidney and the lung in the decreasing order (Langham et al., 1980). The deposition of Pu attributed to the global fall-out from nuclear tests is the highest in the liver then the bone and the lung in the decreasing order in the human body (Taylor, 1995). Pu distribution in the Nagasaki victims was characteristic, that is, radioactivity concentration was not significantly different among organs examined. This suggests that the Nagasaki victims ingested Pu via various ways as well as the lungs, and that soluble Pu was redistributed via blood stream. Pu radioactivity concentration in the Nagasaki victims exposed indoors tended to be lower than those exposed outdoors, indicating that buildings provided shielding from dust containing plutonium. For Nagasaki case 1, except for gender, details were unknown and deceased only 16 days after the bombing, suggesting that he was severely injured immediately. Histological findings of the hilar lymph node were found in the pathological record of Nagasaki case 1, we therefore, assumed that they were hilar lymph nodes. Even soluble forms of Pu accumulate in macrophages within 14 days after intratracheal administration (Van der Meeren et al., 2012). Alpha-tracks were not concentrated in macrophages but were emitted from parenchymal cells in the Nagasaki victims. These suggest that Pu in the Nagasaki victims has long maintained water solubility. As a result, significant amount of Pu radioactivity was observed in the brain of Nagasaki case 1, more than in the liver and in the lymph node. These suggest that hematogenous Pu redistribution started immediately after the ingestion even into the brain through the blood brain barrier. Pu concentration in the bone was the highest among organs examined in Nagasaki case 5, suggesting that the case was outdoors and that a major part of the Pu directly entered the bloodstream through wounds.

Total external dose estimated by DS02 was 83.0 Gy at 500 m of ground distance from the hypocenter (Nagasaki case 3, 4 and 5) and 21.5 Gy at 800 m (Case 2) (https://www.rerf.or.jp/glossary_e/kermat4.htm browsed on Dec. 11, 2017.). In the present study, the organ dose was at most 0.14 mGy during her surviving period in the lung of case 6. Assuming that the distribution of Pu did not change, the 50-year accumulated dose would be 20.2 mGy in the bone marrow of Nagasaki case 3. A clear pattern of increasing risk with dose level was observed above 10 mGy in Mayak workers (Gillies et al., 2017). This indicates that internal dose at the organ level of the Nagasaki victims was extremely low and the effects of Pu exposure on the Nagasaki victims were negligible compared with external dose. The frequency of Thortrast liver cancer, typical internal exposure-induced cancer by alpha-particles, is linearly associated with total dose up to 6.5 Gy (Fukumoto, 2014). Nevertheless, the incubation period from Thorotrast administration to cancer induction is fairly constant irrespective of the deposited amount of thorium and the minimal exposure dose observed for cancer induction was 1.5 Gy at the organ level with the minimal incubation period of 20 years. These facts indicate that the frequency of cancer induction by internal exposure is not merely dependent on organ dose but a consequence of complex events, which cannot be understood without considering the uneven distribution of radionuclides in the affected organ, the number of hits per unit time of alpha-particles, bystander effect and dynamic biological response to radiation (Yamamoto et al., 2009; Fukumoto, 2014). The largest absorbed dose of a cell nucleus penetrated by an alpha-particle from Pu was 3.89 Gy for the vascular endothelial cells. Angiosarcoma derived from vascular endothelial cells is characteristic to Thorotrast laden livers and cancers of other organs as well as liver cancer are induced in Thorotrast patients (Mori et al., 1999). It is relevant and meaningful to analyze the relationship of the impact of internal exposure at the cellular level and organ dose.

In the present study, autoradiographic analysis of 70-year old pathological specimens has revealed Pu in various body tissues from even a limited number of short-term survivors of Nagasaki hibakusha. The observed organ dose was well below levels at which they would have contributed significantly to excess cancer incidences. Extensive studies of hibakusha have shown increased cancer risks at multiple anatomical sites (Nakashima et al., 2007). In addition to the specimens utilized in this study, a tumor tissue bank of Nagasaki hibakusha and Thorotrast patients is maintained at Nagasaki University (Miura et al., 2015, http://www2.idac.tohoku.ac.jp/misc/thorotrast/index%20english.html). The steady comprehensive studies comparing between these valuable archives will make a significant contribution to a fuller understanding of the long-term effects of internal radiation exposure.

## 4 Material & methods

### 4.1 Study design

**Fig. 4:**
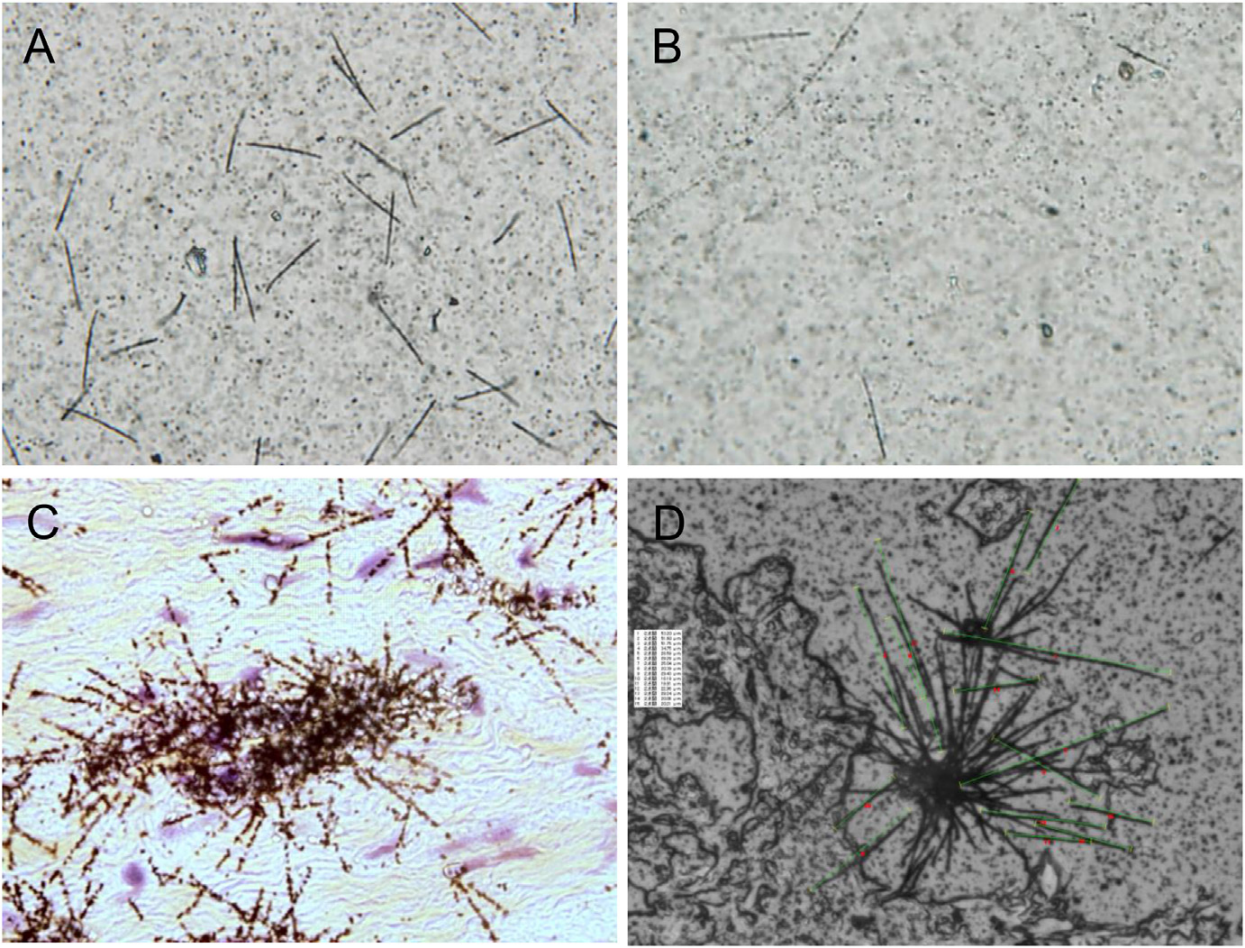
Autoradiographs of alpha particles from samples. (A)^239, 240^Pu in Nagasaki soil by nuclear emulsion method; Ground surface soil collected in 1979 from the Nishiyama area in Nagasaki City, (B) electroplated ^210^Po sample by nuclear emulsion method, (C) H&E stain after photo emulsion method and (D) nuclear emulsion method of alpha tracks from tissues in Thorotrast injected person. Male, 54 years of age, died of cholangiocarcinoma. Thorotrast, a commercial product of 25 % colloidal solution of a natural alpha-particle emitter, thorium dioxide, used as a contrast medium in diagnostic roentgenography for ∼25 years (Fukumoto, 2014).

Paraffin-embedded autopsy specimens of Nagasaki victims collected within 5 months from late August 1945 were returned to Japan in 1973 from the U.S. Armed Forces Institute of Pathology (Okamoto et al., 1973). After a preliminary review, tissues from 170 Nagasaki victims were found to be sufficiently well preserved to justify detailed histologic study. However, only 10 cases were found to be adequate for the present study provided with the location within 1 km from the hypocenter, the date of death within 5 months from the bombing and possible protective factors. From them, 7 well preserved cases were selected after the histological sections were stained with hematoxylin and eosin (H&E) (Table 1). Non-exposed cases (controls) were chosen among individuals dead of disease unrelated to radiation and autopsied during 1952–1954 at hospitals 30 km or 130 km distant from the hypocenter. Paraffin-embedded specimens of a Thorotrast patient were used as a calibration standard for track lengths with known energy alpha particles (Yamamoto et al., 2009) (Fig. 4).

**Table 1:** Case profile.

Case	Age	Gender	Distance	Death Date	Protection
			(km)	(day)	
Nagasaki A bomb case					
1	uk	M	uk	16	uk
2	21	M	0.8	38	indoors
3	60	F	0.5	68	outdoors in open
4	43	M	0.5	78	Japanese wooden building (indoors)
5	23	M	0.5	87	uk
6	33	F	uk	125	burned under the fallen house
7	77	F	1	144	sitting behind tree in open
Mean	(42.8)		(0.66)	(79.4)	
Control					
1	31	F	10	2480	
2	37	F	210	2643	
3	26	M	120	2731	
4	54	M	110	2774	
5	60	F	17	2840	
6	31	M	47	3160	
7	28	M	18	3197	
Mean	(37.9)		(76.1)	(2868)	

uk: unknown.

### 4.2 Procedures

Paraffin-embedded specimens were sectioned at 4 μm thickness and were used for autoradiography. Alpha-tracks in the tissue specimens were detected by the photo emulsion dipping method (Rogers, 1967) and the nuclear emulsion contact method. For the dipping method, sections of tissues were mounted on glass slides, and the unstained sections were dipped in undiluted NTB3 liquid photographic emulsion (Eastman Kodak Co.). For the contact method, sections of the tissues mounted on the glass slides were covered with the nuclear emulsion sheet (ILFORD SCIENTIFIC PRODUCT L4 PLATES). The sections were exposed for 6 months. After development, the slides were stained with H&E. Particle track lengths were measured using a standard optical microscope at 1000x magnification (BIOREVO, BZ-9000, KEYENCE). Autoradiography was undertaken in triplicates. At least two individuals participated in counting the particle tracks independently and confirmed the number of tracks if a difference occurred. In total, 212 slides were scored in which 3,570 alpha-tracks in total were counted.

### 4.3 Estimation of radioactivity concentration

#### Calculating alpha track length on photo emulsion

**Fig. 5:**
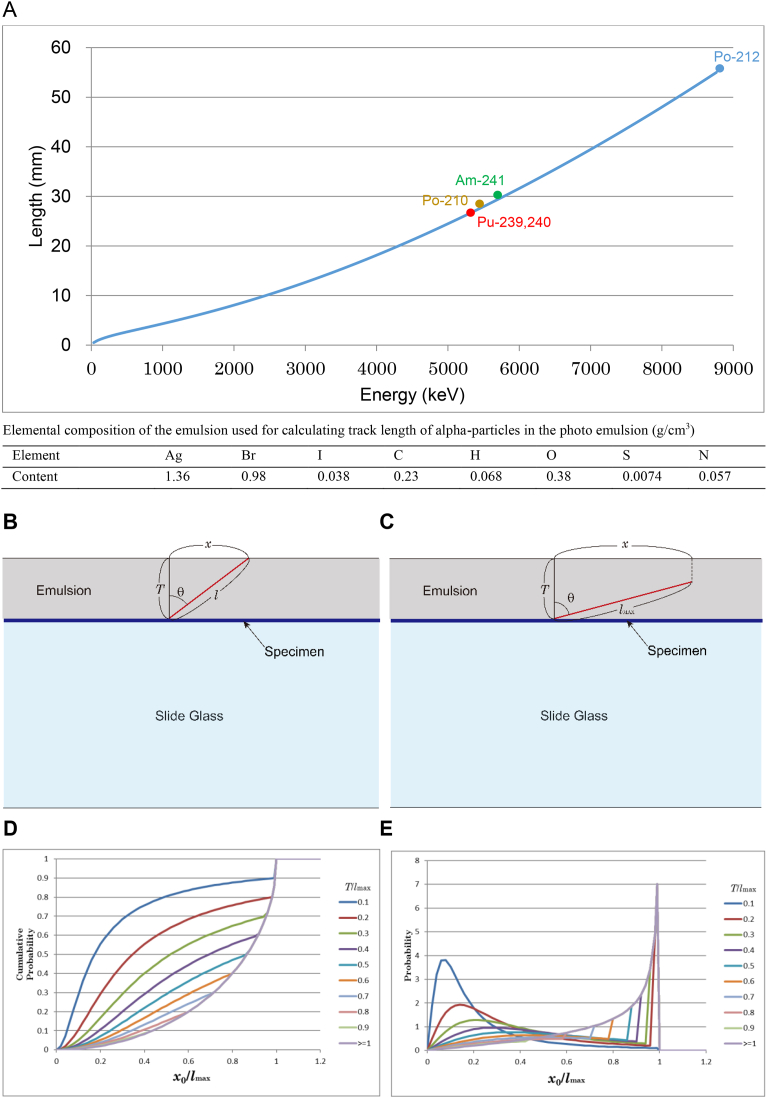
(A) Track length on photo emulsion calculated. (B) $T<l_{MAX}$ and $x_{0}<\sqrt{l_{MAX}^{2}-T^{2}}$. (C) $T\geq l_{MAX}$ or $x_{0}\geq \sqrt{l_{MAX}^{2}-T^{2}}$. (D) Cumulative probability Eq. (4) versus $\frac{x_{0}}{l_{\text{max}}}$ . The value depends on $\frac{T}{l_{\text{max}}}$. (E) Differential probability of Eq. AP-4 versus $\frac{x_{0}}{l_{\text{max}}}$. The value was simply obtained through difference method. A sharp peak appears near $\frac{x_{0}}{l_{\text{max}}}=1$ when $\frac{T}{l_{\text{max}}}$ is small.

The relation between energy and track length of alpha-particles was calculated by continuously slowing down approximation (CSDA) using the helium stopping powers for each element in the emulsion deduced from Zeigler's semi-empirical formula (Zeigler, 1977). The length of the track of a 8.787 MeV alpha-particle emitted from ^212^Po of the thorium series was used for calibration. Fig. 5A is the calculated track length. The elemental composition is used for the calculation. The composition was estimated as follows.

Photo emulsion is composed of dry emulsion and moisture content and was referred to in the data of Ilford nuclear emulsion in Norris and Woodruff (1955). The emulsion used was not made by Ilford, but differences between manufacturers are very small according to the article. Content of gelatin was not clear, and the content was adjusted so that the track length of Po-212 8.785 MeV alpha becomes the length of experimental value 55 μm. Pu-239,240 is clearly distinguished from other alpha-emitters (Po-210; 5.304 MeV, Am-241; 5.485 MeV, Po-212; 8.785 MeV).

The magnitude of the stopping powers is largely different between elements, but the relative energy dependence is almost the same between elements. Therefore, the effect of any error of the estimated composition to the calculated energy-track length relation is likely negligible.

#### Probability distribution of observed track length

Probability distribution of the observed track length was represented as differential probability (Fig. 5E) and cumulative probability (Fig. 5D) deduced from the distribution of the alpha-track length (Fig. 5A–E). Fig. 5B and C show geometrical conditions of an alpha particle emitted from a tissue specimen between a slide glass and photo emulsion layer. The alpha particle penetrates the emulsion in Fig. 5B and the alpha particle stops in the emulsion in Fig. 5C. *T* is thickness of the emulsion, *x* is the observed length of the alpha track, *l* is the true length of the alpha track, *l*_MAX_ is the maximum of *l* and it is determined by the energy of the alpha particle (Fig. 5A), and θ is the angle of the emission.

Denoting $\text{Ω}\left(\theta_{0}\right)$ the solid angle of the emission with θ smaller than $\theta_{0}$ and $\text{Ω}\left(x_{0}\right)$ the solid angle of the emission with *x* shorter than *x*_0_, from the relation dΩ(θ)=2πsinθdθ,

(1) $$\text{Ω}\left(\theta_{0}\right)=\int_{0}^{\theta_{0}}2\pi \;\sin\;\theta d\theta =2\pi {\left[-\cos\;\theta \right]}_{0}^{\theta_{0}}=2\pi \left(1-\cos\;\theta_{0}\right)\text{.}$$

When $T<l_{MAX}$ and $x_{0}<\sqrt{l_{MAX}^{2}-T^{2}}$, $\cos\;\theta =\frac{T}{\sqrt{x_{0}^{2}+T^{2}}}$. Therefore,

(2) $$\text{Ω}\left(x_{0}\right)=2\pi \left(1-\frac{T}{\sqrt{x_{0}^{2}+T^{2}}}\right)$$

When $T\geq l_{MAX}$ or $x_{0}\geq \sqrt{l_{MAX}^{2}-T^{2}}$, $x_{0}=l_{MAX}\;\sin\;\theta$. Therefore,

(3) $$\text{Ω}\left(x_{0}\right)=2\pi \left(1-\cos\;\theta \right)=2\pi \left(1-\sqrt{1-{\left(\frac{x_{0}}{l_{MAX}}\right)}^{2}}\right)$$

The solid angle that an alpha particle is emitted into the emulsion is 2π. Therefore, the probability that an observed alpha-particle track length is shorter than $x_{0}$ is $\frac{\text{Ω}\left(x_{0}\;\right)}{2\pi }$. Therefore, the cumulative probability from zero to $x_{0}$ is

(4) $$P\left(x_{0}\right)=\{\begin{array}{cc} 1-\frac{T}{\sqrt{x_{0}^{2}+T^{2}}} & \left(T<l_{MAX}\;\text{and}\;x_{0}<\sqrt{l_{MAX}^{2}-T^{2}}\right)\\ 1-\sqrt{1-{\left(\frac{x_{0}}{l_{MAX}}\right)}^{2}} & \left(T\geq l_{MAX}\;\text{or}\;x_{0}\geq \sqrt{l_{MAX}^{2}-T^{2}}\right) \end{array}$$

Eq. (4) is graphically shown in Fig. 5D. Differential probability is shown in Fig. 5E. A sharp peak appears near $\frac{x_{0}}{l_{\text{max}}}=1$ when $\frac{T}{l_{\text{max}}}$ is small.

#### Radioactivity

(5) $$\text{From}\;\text{Eq}\text{.}\;\left(\text{4}\right)\text{,}\;P\left(x>x_{0}\right)=1-P\left(x_{0}\right)$$

Therefore, the probability that an alpha particle emission (including toward the back side) makes a track which is observed longer than *x*_0_ is:

(6) $$Eff=\frac{1}{2}P\left(x>x_{0}\right)=\frac{1-P\left(x_{0}\right)}{2}$$

Radioactivity per volume *A*_*v*_ are represented as

(7) $${\text{A}}_{V}=\frac{n}{Eff\cdot t\cdot V}$$

V=mSU

*n*: total number of tracks longer than *x*.
*V*: total volume of sample tissue.
*t*: time of exposure.
*m*: number of samples
*S*: area of samples
*U*: thickness of a slice
*Eff* was evaluated 0.0657255 and the Pu radioactivity per unit volume, *Av* was deduced using the following equation:

(8) $$Av=\frac{N}{0.0657255\times t}\text{,}$$

*N*: the observed number of alpha-tracks per volume whose length is in the area of the plutonium peak (25.25–26.25 μm) (Fig. 2, Tables 2 and 3). The value 0.0657255 was determined from the notion that *T*/*l*_max_ was approximately 0.2 and *x*_0_/*l*_max_ was 25.25/25.5 where 25.25 μm is the lower boundary of the plutonium peak area, the maximum length *l*_max_ = 25.5 μm and the thickness of emulsion *T* = 5 μm (Eqs. (4), (5), (6), (7), Fig. 5D). The maximum length observed was a little longer than 25.5 μm because of the variation observed around the peak.

**Table 2:** Numerical data of Fig. 2.

Length (μm)	A bomb			Control			Thorotrast		
	Frequency	Differential probability	Cumulative probability	Frequency	Differential probability	Cumulative probability	Frequency	Differential probability	Cumulative probability
0.5	0	0.000	0.000	0	0.000	0.000	0	0.000	0.000
1.0	3	0.015	0.015	2	0.008	0.008	0	0.000	0.000
1.5	3	0.015	0.030	6	0.024	0.032	0	0.000	0.000
2.0	4	0.020	0.050	2	0.008	0.040	0	0.000	0.000
2.5	9	0.045	0.095	16	0.063	0.103	0	0.000	0.000
3.0	8	0.040	0.134	7	0.028	0.130	0	0.000	0.000
3.5	4	0.020	0.154	12	0.047	0.178	1	0.002	0.002
4.0	3	0.015	0.169	3	0.012	0.190	0	0.000	0.002
4.5	2	0.010	0.179	2	0.008	0.198	0	0.000	0.002
5.0	5	0.025	0.204	13	0.051	0.249	21	0.033	0.035
5.5	5	0.025	0.229	6	0.024	0.273	10	0.016	0.050
6.0	1	0.005	0.234	4	0.016	0.289	17	0.027	0.077
6.5	0	0.000	0.234	0	0.000	0.289	13	0.020	0.097
7.0	2	0.010	0.244	2	0.008	0.296	13	0.020	0.118
7.5	5	0.025	0.269	15	0.059	0.356	55	0.086	0.204
8.0	6	0.030	0.299	10	0.040	0.395	12	0.019	0.223
8.5	1	0.005	0.303	4	0.016	0.411	8	0.013	0.235
9.0	2	0.010	0.313	8	0.032	0.443	23	0.036	0.272
9.5	1	0.005	0.318	1	0.004	0.447	5	0.008	0.279
10.0	18	0.090	0.408	22	0.087	0.534	62	0.097	0.377
10.5	3	0.015	0.423	1	0.004	0.538	11	0.017	0.394
11.0	2	0.010	0.433	5	0.020	0.557	8	0.013	0.407
11.5	2	0.010	0.443	1	0.004	0.561	14	0.022	0.429
12.0	1	0.005	0.448	1	0.004	0.565	9	0.014	0.443
12.5	9	0.045	0.493	22	0.087	0.652	24	0.038	0.480
13.0	1	0.005	0.498	6	0.024	0.676	8	0.013	0.493
13.5	5	0.025	0.522	3	0.012	0.688	10	0.016	0.509
14.0	0	0.000	0.522	5	0.020	0.708	6	0.009	0.518
14.5	2	0.010	0.532	0	0.000	0.708	7	0.011	0.529
15.0	17	0.085	0.617	22	0.087	0.794	38	0.060	0.589
15.5	0	0.000	0.617	0	0.000	0.794	6	0.009	0.598
16.0	4	0.020	0.637	1	0.004	0.798	11	0.017	0.615
16.5	1	0.005	0.642	1	0.004	0.802	13	0.020	0.636
17.0	0	0.000	0.642	3	0.012	0.814	6	0.009	0.645
17.5	6	0.030	0.672	5	0.020	0.834	31	0.049	0.694
18.0	0	0.000	0.672	5	0.020	0.854	6	0.009	0.703
18.5	2	0.010	0.682	2	0.008	0.862	4	0.006	0.710
19.0	0	0.000	0.682	1	0.004	0.866	10	0.016	0.725
19.5	0	0.000	0.682	1	0.004	0.870	4	0.006	0.732
20.0	13	0.065	0.746	14	0.055	0.925	24	0.038	0.769
20.5	0	0.000	0.746	0	0.000	0.925	2	0.003	0.772
21.0	1	0.005	0.751	0	0.000	0.925	7	0.011	0.783
21.5	3	0.015	0.766	0	0.000	0.925	5	0.008	0.791
22.0	0	0.000	0.766	0	0.000	0.925	3	0.005	0.796
22.5	4	0.020	0.786	1	0.004	0.929	18	0.028	0.824
23.0	1	0.005	0.791	2	0.008	0.937	1	0.002	0.826
23.5	0	0.000	0.791	2	0.008	0.945	5	0.008	0.834
24.0	0	0.000	0.791	3	0.012	0.957	5	0.008	0.841
24.5	1	0.005	0.796	0	0.000	0.957	5	0.008	0.849
25.0	2	0.010	0.806	1	0.004	0.960	36	0.057	0.906
25.5	39	0.194	1.000	7	0.028	0.988	2	0.003	0.909
26.0	0	0.000	1.000	2	0.008	0.996	3	0.005	0.914
26.5	0	0.000	1.000	0	0.000	0.996	3	0.005	0.918
27.0	0	0.000	1.000	0	0.000	0.996	0	0.000	0.918
27.5	0	0.000	1.000	1	0.004	1.000	4	0.006	0.925
28.0	0	0.000	1.000	0	0.000	1.000	5	0.008	0.932
28.5	0	0.000	1.000	0	0.000	1.000	0	0.000	0.932
29.0	0	0.000	1.000	0	0.000	1.000	4	0.006	0.939
29.5	0	0.000	1.000	0	0.000	1.000	2	0.003	0.942
30.0	0	0.000	1.000	0	0.000	1.000	5	0.008	0.950
30.5	0	0.000	1.000	0	0.000	1.000	2	0.003	0.953
31.0	0	0.000	1.000	0	0.000	1.000	2	0.003	0.956
31.5	0	0.000	1.000	0	0.000	1.000	1	0.002	0.958
32.0	0	0.000	1.000	0	0.000	1.000	2	0.003	0.961
32.5	0	0.000	1.000	0	0.000	1.000	3	0.005	0.965
33.0	0	0.000	1.000	0	0.000	1.000	0	0.000	0.965
33.5	0	0.000	1.000	0	0.000	1.000	1	0.002	0.967
34.0	0	0.000	1.000	0	0.000	1.000	1	0.002	0.969
34.5	0	0.000	1.000	0	0.000	1.000	1	0.002	0.970
35.0	0	0.000	1.000	0	0.000	1.000	4	0.006	0.976
35.5	0	0.000	1.000	0	0.000	1.000	0	0.000	0.976
36.0	0	0.000	1.000	0	0.000	1.000	2	0.003	0.980
36.5	0	0.000	1.000	0	0.000	1.000	0	0.000	0.980
37.0	0	0.000	1.000	0	0.000	1.000	1	0.002	0.981
37.5	0	0.000	1.000	0	0.000	1.000	0	0.000	0.981
38.0	0	0.000	1.000	0	0.000	1.000	0	0.000	0.981
38.5	0	0.000	1.000	0	0.000	1.000	0	0.000	0.981
39.0	0	0.000	1.000	0	0.000	1.000	0	0.000	0.981
39.5	0	0.000	1.000	0	0.000	1.000	0	0.000	0.981
40.0	0	0.000	1.000	0	0.000	1.000	2	0.003	0.984
40.5	0	0.000	1.000	0	0.000	1.000	0	0.000	0.984
41.0	0	0.000	1.000	0	0.000	1.000	2	0.003	0.987
41.5	0	0.000	1.000	0	0.000	1.000	0	0.000	0.987
42.0	0	0.000	1.000	0	0.000	1.000	0	0.000	0.987
42.5	0	0.000	1.000	0	0.000	1.000	0	0.000	0.987
43.0	0	0.000	1.000	0	0.000	1.000	0	0.000	0.987
43.5	0	0.000	1.000	0	0.000	1.000	1	0.002	0.989
44.0	0	0.000	1.000	0	0.000	1.000	0	0.000	0.989
44.5	0	0.000	1.000	0	0.000	1.000	0	0.000	0.989
45.0	0	0.000	1.000	0	0.000	1.000	2	0.003	0.992
45.5	0	0.000	1.000	0	0.000	1.000	0	0.000	0.992
46.0	0	0.000	1.000	0	0.000	1.000	0	0.000	0.992
46.5	0	0.000	1.000	0	0.000	1.000	0	0.000	0.992
47.0	0	0.000	1.000	0	0.000	1.000	0	0.000	0.992
47.5	0	0.000	1.000	0	0.000	1.000	1	0.002	0.994
48.0	0	0.000	1.000	0	0.000	1.000	0	0.000	0.994
48.5	0	0.000	1.000	0	0.000	1.000	0	0.000	0.994
49.0	0	0.000	1.000	0	0.000	1.000	0	0.000	0.994
49.5	0	0.000	1.000	0	0.000	1.000	0	0.000	0.994
50.0	0	0.000	1.000	0	0.000	1.000	2	0.003	0.997
50.5	0	0.000	1.000	0	0.000	1.000	0	0.000	0.997
51.0	0	0.000	1.000	0	0.000	1.000	1	0.002	0.998
51.5	0	0.000	1.000	0	0.000	1.000	0	0.000	0.998
52.0	0	0.000	1.000	0	0.000	1.000	0	0.000	0.998
52.5	0	0.000	1.000	0	0.000	1.000	1	0.002	1.000
53.0	0	0.000	1.000	0	0.000	1.000	0	0.000	1.000
53.5	0	0.000	1.000	0	0.000	1.000	0	0.000	1.000
54.0	0	0.000	1.000	0	0.000	1.000	0	0.000	1.000
54.5	0	0.000	1.000	0	0.000	1.000	0	0.000	1.000
55.0	0	0.000	1.000	0	0.000	1.000	0	0.000	1.000
55.5	0	0.000	1.000	0	0.000	1.000	0	0.000	1.000
Total	201			253			637

**Table 3:** Track number in ^239^Pu peak area from each sample.

Case	Tracks/time (s^−1^)	SE	Case	Tracks/time (s^−1^)	SE
A1-bladder	3.59E-07	8.71E-08	A5-small intestine	5.43E-08	5.43E-08
A1-brain	4.44E-07	8.38E-08	A5-spleen	2.24E-08	2.24E-08
A1-colon	6.34E-08	6.34E-08	A5-stomach	1.63E-07	9.41E-08
A1-heart	1.9E-07	1.1E-07	A5-testis	8.95E-08	4.47E-08
A1-kidney	6.34E-08	6.34E-08	A5-trachea	2.24E-07	7.07E-08
A1-liver	7.92E-08	3.54E-08	A5-vessel	5.43E-08	5.43E-08
A1-lymph node	9.51E-08	3.88E-08	A6-heart	3.17E-07	6.34E-08
A1-pancreas	9.51E-08	3.88E-08	A6-kidney	6.13E-07	1.14E-07
A1-stomach	0	0	A6-liver	3.55E-07	6.71E-08
A2-adrenal gland	6.71E-08	3.87E-08	A6-lung	7.82E-07	1.29E-07
A2-colon	5.43E-08	5.43E-08	A6-spleen	1.58E-07	5.01E-08
A2-kidney	4.47E-08	3.16E-08	A7-adrenal gland	2.24E-08	2.24E-08
A2-liver	9.92E-08	4.05E-08	A7-bile duct	7.61E-08	5.38E-08
A2-lung	0	0	A7-bone marrow	2.91E-07	8.07E-08
A2-prostate	6.71E-08	3.87E-08	A7-kidney	1.79E-07	6.33E-08
A2-spleen	0	0	A7-liver	7.13E-08	4.12E-08
A2-testis	1.09E-07	7.68E-08	A7-lung	1.12E-07	5E-08
A3-bone marrow	2.38E-07	6.14E-08	A7-spleen	0	0
A3-kidney	3.8E-07	1.55E-07	A7-stomach	4.47E-08	3.16E-08
A3-liver	5.7E-07	1.9E-07	C1-liver	0	0
A3-lung	1.58E-07	5.01E-08	C1-lung	0	0
A3-lymph node	4.23E-08	2.99E-08	C1-spleen	0	0
A3-spleen	4.44E-07	1.68E-07	C1-striated muscle	0	0
A4-adrenal gland	5.43E-08	5.43E-08	C1-thyroid	0	0
A4-colon	1.12E-07	5E-08	C2-lung	0	0
A4-kidney	1.09E-07	7.68E-08	C3-kidney	0	0
A4-liver	0	0	C3-liver	0	0
A4-lung	2.24E-08	2.24E-08	C3-lung	0	0
A4-spleen	0	0	C3-pancreas	0	0
A4-stomach	2.24E-08	2.24E-08	C3-spleen	0	0
A5-artery	1.34E-07	5.48E-08	C4-bladder	0	0
A5-bile duct	0	0	C4-heart	0	0
A5-bone	1.16E-07	4.37E-08	C4-kidney	0	0
A5-bone marrow	1.79E-07	6.33E-08	C4-liver	0	0
A5-colon	6.71E-08	3.87E-08	C4-lung	0	0
A5-diaphragm	1.09E-07	7.68E-08	C4-spleen	0	0
A5-esophagus	1.63E-07	9.41E-08	C5-heart	0	0
A5-gallbladder	2.01E-07	6.71E-08	C5-lung	0	0
A5-heart	2.24E-07	7.07E-08	C5-spleen	0	0
A5-ileocecal valve	1.12E-07	5E-08	C6-kidney	0	0
A5-kidney	4.89E-07	1.63E-07	C6-lung	6.34E-08	6.34E-08
A5-liver	1.57E-07	5.92E-08	C6-spleen	0	0
A5-lung	2.01E-07	6.71E-08	C7-heart	0	0
A5-lymph node	1.57E-07	5.92E-08	C7-kidney	0	0
A5-pancreas	3.36E-07	8.66E-08	C7-liver	0	0
A5-salivary gland	1.09E-07	7.68E-08	C7-spleen	0	0
A5-seminal vesicle	8.95E-08	4.47E-08			

#### Absorbed dose

Absorbed dose rate $\dot{D}$ was deduced using the following equation:

(9) $$\dot{D}=\frac{A\bar{E}}{\rho }\text{,}$$

(10) $$D=\int_{t_{0}}^{t_{1}}\dot{D}\left(t\right)dt$$

(11) $$\dot{D}\left(t\right)={\left(\frac{1}{2}\right)}^{\frac{t-t_{0}}{T}}\dot{D}\left(t_{0}\right)$$

Where, *A* is the radioactivity per unit volume, $\bar{E}$ is the mean energy of alpha-particles emitted from ^239^Pu, ρ is the mass density of the tissue, D is the absorbed dose, $t_{0}$ is the time of intake, $t_{1}$ is the time absorbed dose is evaluated ($t_{0}$ + (survival time)), and T is the biological half-life (50 years).

**Fig. 6:**
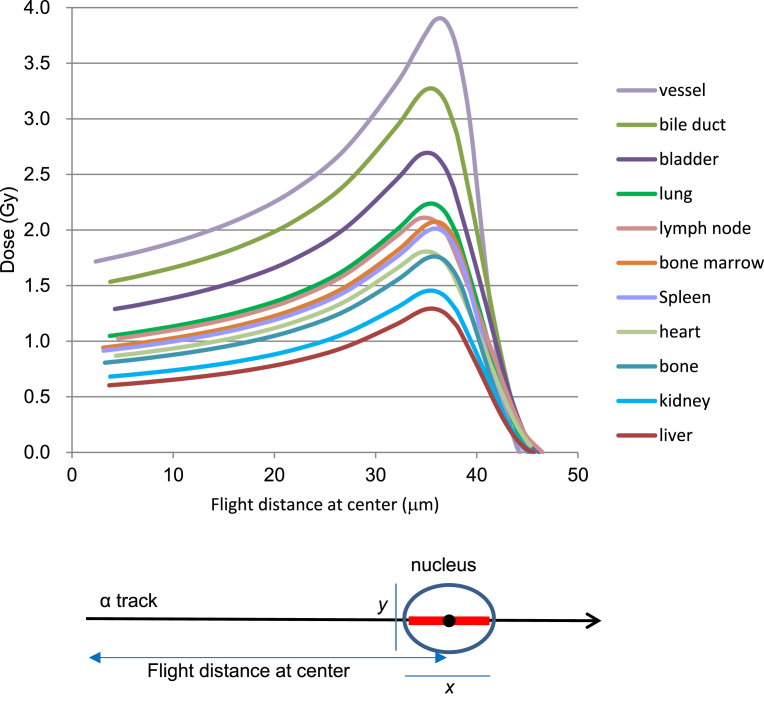
Absorbed energy from an alpha-particle passing an ellipsoidal cell nucleus. Shape of the cell nucleus was assumed to be spheroid. The nuclear size in the cell (long axis *x* and short axis *y*) of each organ was measured under a microscope (Table 4). Absorbed energy from alpha-particles of various energies was calculated using empirical formula of alpha-particle range and nuclear size. When variations of LET along the alpha particle path can be neglected, absorbed energy *E* from an alpha-particle passing an ellipsoidal cell nucleus along the long axis is given by E=Lx, where, *L* is LET and *x* is the length of the long axis. Volume of the nucleus is given by $V=\frac{4}{3}\pi \left(\frac{x}{2}\right){\left(\frac{y}{2}\right)}^{2}=\frac{1}{6}\pi xy^{2}\text{,}$ when the length of the two shorter axes is equal and are denoted *y*. The mass of the nucleus is m=ρV where, ρ is the density of the nucleus, and the absorbed dose of the nucleus is given by $D=\frac{E}{m}=\frac{Lx}{\rho V}=\frac{6L}{\pi y^{2}\rho }$. Therefore, when variations of LET along the alpha particle path in the cell nucleus can be neglected, the absorbed dose is determined by the value *y*.

In order to consider the biological effect of internal exposure by alpha-particles, absorbed dose of a cell nucleus was estimated. Assuming the nucleus is ellipsoidal, long axis and short axis of cells in each organ were measured under a microscope. Absorbed energy from alpha-particles of varying energies was calculated using Zeigler's semi-empirical formula of alpha-particle range and the nuclear size (Fig. 6, Table 4).

**Table 4:** Nuclear size parameters used in Fig. 6

Organs	Long axis (μm)	Short axis (μm)
bile duct	7.57	4.18
bladder	8.49	4.57
bone	6.53	5.74
bone marrow	6.18	5.31
heart	8.70	5.57
kidney	7.57	6.27
liver	7.38	6.66
lung	7.50	5.06
lymph node	9.14	5.13
spleen	6.24	5.39
vessel	4.70	3.92

### 4.4 Statistical analysis

Errors of radioactivity were estimated with the assumption that the number of alpha-particles emitted follows the Poisson distribution.

## Declarations

### Author contribution statement

Kazuko Shichijo: Conceived and designed the experiments; Performed the experiments; Analyzed and interpreted the data; Contributed reagents, materials, analysis tools or data; Wrote the paper.

Toshihiro Takatsuji: Conceived and designed the experiments; Analyzed and interpreted the data; Wrote the paper.

Manabu Fukumoto: Analyzed and interpreted the data; Wrote the paper.

Masahiro Nakashima: Contributed reagents, materials, analysis tools or data.

Mutsumi M Matsuyama: Performed the experiments.

Ichiro Sekine: Conceived and designed the experiments; Contributed reagents, materials, analysis tools or data.

### Funding statement

This work was supported by Grants-in-Aid for Scientific Research (C) (No. 23510064) (KAKENHI), Japan. This work was also partly supported by the Cooperative Research Project Program of Joint Usage/Research Center at the Institute of Development, Aging and Cancer, Tohoku University and the Program of the Network-type Joint Usage/Research Center for Radiation Disaster Medical Science of Hiroshima University, Nagasaki University and Fukushima Medical University.

### Competing interest statement

The authors declare no conflict of interest.

### Additional information

No additional information is available for this paper.
